# Intensive Cytokine induction in Pandemic H1N1 Influenza Virus Infection Accompanied by Robust Production of IL-10 and IL-6

**DOI:** 10.1371/journal.pone.0028680

**Published:** 2011-12-09

**Authors:** Xuelian Yu, Xi Zhang, Baihui Zhao, Jiayu Wang, Zhaokui Zhu, Zheng Teng, Junjie Shao, Jiaren Shen, Ye Gao, Zhengan Yuan, Fan Wu

**Affiliations:** 1 Microbiology Laboratory, Shanghai Municipal Center for Disease Control and Prevention, Shanghai, People's Republic of China; 2 Shanghai Municipal Center for Disease Control and Prevention, Shanghai, People's Republic of China; The Scripps Research Institute, United States of America

## Abstract

**Background:**

The innate immune system is the first line of defense against viruses by inducing expression of cytokines and chemokines. Many pandemic influenza H1N1 virus [P(H1N1)] infected severe cases occur in young adults under 18 years old who were rarely seriously affected by seasonal influenza. Results regarding host cytokine profiles of P(H1N1) are ambivalent. In the present study we investigated host cytokine profiles in P(H1N1) patients and identified cytokines related to disease severity.

**Methods and Principal Findings:**

We retrieved 77, 59, 26 and 26 sera samples from P(H1N1) and non-flu influenza like illness (non-ILIs) cases with mild symptoms (mild patients), P(H1N1) vaccinees and healthy individuals, respectively. Nine and 16 sera were from hospitalized P(H1N1) and non-ILIs patients with severe symptoms (severe patients). Cytokines of IL-1, IL-2, IL-4, IL-5, IL-6, IL-8, IL-10, IL-12, IFN-γ and TNF-α were assayed by cytokine bead array, IL-17 and IL-23 measured with ELISA. Mild P(H1N1) patients produced significantly elevated IL-2, IL-12, IFN-γ, IL-6, TNF-α, IL-5, IL-10, IL-17 and IL-23 versus to healthy controls. While an overwhelming IL-6 and IL-10 production were observed in severe P(H1N1) patients. Higher IL-10 secretion in P(H1N1) vaccinees confirmed our observation that highly increased level of sera IL-6 and IL-10 in P(H1N1) patients may lead to disease progression.

**Conclusion and Significance:**

A comprehensive innate immune response was activated at the early stage of P(H1N1) infection with a combine Th1/Th2/Th3 cytokines production. As disease progression, a systemic production of IL-6 and IL-10 were observed in severe P(H1N1) patients. Further analysis found a strong correlation between IL-6 and IL-10 production in the severe P(H1N1) patients. IL-6 may be served as a mediator to induce IL-10 production. Highly elevated level of sera IL-6 and IL-10 in P(H1N1) patients may lead to disease progression, but the underlying mechanism awaits further detailed investigations.

## Introduction

Pandemic H1N1/09A [P(H1N1)] of swine origin has led to a global spread of pandemic in 2009 and 2010. This new pandemic strain is of particular concern because of its efficient person-to-person transmission and reassortments of potentially increased virulence. Experiments have indicated that P(H1N1) increases morbidity in human [1], [2], [3]. Since P(H1N1) can persist in the human population, it can potentially cause even more severe clinical consequences.

The innate immune system, the first line of defense against invading viruses, involves two types of cytokine responses: a proinflammatory response and an antiviral response. Inflammatory cytokines and chemokines play a role in the pathogenesis of virus infection in animals and humans [4]. Humans infected with highly virulent influenza viruses together with aberrant and excessive cytokine production are linked to morbidity and mortality. Over-production of specific inflammatory cytokines, such as the tumor necrosis factor (TNF)-a, interleukin (IL)-1, IL-6 and IL-10, as well as the polymorphonuclear neutrophil CC chemokine (chemokine) IL-8, is the hallmark of viral infection [5]. A cytokine-mediated inflammatory response, characterized by hyper-induction of proinflammatory cytokine production, also known as hypercytokinemia, has been well documented as an important player in the disease progression and the ultimate death of patients infected by most seasonal influenza. Excessive cytokines (IL-6, TNF-a and IFN-γ), or elevated cytokine (IL-6, IL-12, and IFN-γ) levels have been observed in community acquired acute seasonal influenza A illness [6], or in severe seasonal influenza patients [7]. However, the innate immune response related to P(H1N1) virus infection and the role of most of the cytokines in relation to the disease severity remain thus far unclear.

Results from previous studies regarding the host cytokine profile in cell lines or patients infected with P(H1N1) are ambivalent. Österlund et al [8] reported a poor proinflammatory cytokine gene expression in human monocyte-derived dentritic cells (DCs) and macrophages infected with P(H1N1). Mukherjee et al [9] found that the expression of IL-8 and IL-4 was not induced in the A549 cells infected with P(H1N1). A robust Th17 and Th1 cytokine secretion was reported from severe patients infected with P(H1N1) [10]. Therefore, the conflicting available data from different studies have indicated the complexicity of P(H1N1) virus infection, and warrant the necessity of further studies.

Similar to most strains of seasonal influenza, P(H1N1) infection is self-limiting and uncomplicated with mild clinical symptoms, such as fever and acute upper respiratory track infection symptoms in the great majority of the patients [11]. A small percentage of patients, however, can develop more complicated and severe symptoms, such as pneumonia or acute respiratory distress syndrome, requiring hospitalization [11], [12]. Young adults under the age of 18 years, an age group rarely severely affected by seasonal influenza, have a disproportionally high risk of falling into this small percentage [11], [12], [13]. Surveillance data in Japan also reported that most cases of P(H1 N1) infection with severe symptoms and hospitalizations occurred in individuals aged 5–14 years [14]. Reports from the Centers for Disease Control (CDC) showed that the P(H1N1) virus took a heavier toll among chronically ill children than the seasonal flu usually did. Several hypotheses to explain this phenomenon were advocated, including bacterial infection, down-regulation of type 1 interferon expression, apoptosis and hyperinduction of proinflammatory. Studies have suggested that the severe symptoms of P(H1N1) infection in young adults may be caused by an excessive immune response [15]. However, more data are needed for better understanding.

In an attempt to elucidate the innate immune response to P(H1N1) infection and to gain further insight into cytokine mediated pathogenesis, we retrospectively investigated the expression levels of a panel of serum cytokines and chemokines in different groups of patients and controls, including severe and mild patients infected with P(H1N1), severe and mild patients with non-influenza like illness (non-ILIs), vaccinees immunized with P(H1N1) vaccine and healthy individuals. The results derived from this study may help understand the pathogenic events leading to poor outcomes related to P(H1N1) infection, and design rational strategies for diagnosis and treatment.

## Results

### Patients' characteristics

P(H1N1) cases (77) and non-ILIs patients (59) were all quarantined cases investigated during 2009 influenza pandemic period. The health control individuals (26) were selected from study subjects of 2009 P(H1N1) herd immunity survey. The majority (91%, 70/77) P(H1N1) cases and (88%, 52/59) non-ILIs patients were isolated on the day of fever onset (Chi-sqare test, p = 0.599) and the others on the second day. The age median of P(H1N1) patients and non-ILIs cases were 16 [Inter Quantile Range (IQR): 21–27] and 28 (IQR: 21–39), respectively (p = 0.017, Kruskal Wallis test). Age group of 5∼20 year old consisted of 46.1% (35/77) of total P(H1N1) cases, or 23.7% (14/59) of total non-ILIs patients, p = 0.006, demonstrating a disproportionally high infection rate in these age groups. There was no statistical difference regarding gender distribution in both groups. Compared with the non-ILIs cases whose median body temperature was 37.8°C (IQR: 37.6–38.1), P(H1N1) patients experienced higher fever with a median body temperature of 38.1°C (IQR: 37.8–38.8°C), (p = 0.0004, Kruskal Wallis test), indicating a unique pathogenic nature of P(H1N1).

Both severe P(H1N1) and non-ILIs patients were hospitalized patients from year 2011. Detailed characteristics and clinical manifestation were summarized in Table 1. The age and gender distribution in both groups are similar (p = 0.694) and (p = 0.673). The pathological chest x-ray within 24 hours of onset of the symptoms was taken in all patients. The majority of the patients suffered from fever. The respective body temperature in severe P(H1N1) patients and non-ILIs were 38.5°C (IQR: 37.9–38.8°C) and 38.1°C (IQR: 37.7–39.0°C), p = 0.673), respectively. Cough was common in both groups. However, P(H1N1) cases more frequently had productive cough (p = 0.062), while non-flu ILIs cases had more dry cough (p = 0.008). Severe P(H1N1) patients had higher rate of gastrointestinal symptoms, with 55.5% (5/9) of P(H1N1) and 12.5% (2/16) of non-ILIs patients reported diarrhea, (p = 0.034), respectively.

**Table 1:** Characteristics and medical conditions of severe P(H1N1) patients and non-ILIs cases in Shanghai from year 2011.

Characteristics	No. patients of P(H1N1) (%)	No. patients of non-ILIs (%)	p Value
Confirmed Patients	9	16	
Demographic Information			
Age median (min-max)	2 y(7 m–10 y)	3 y(9 m–12 y)	0.694
Male	6(66.7)	12(75.0)	0.673
Clinical Manifestation			
†BT (95% CI)	38.5(37.9–38.8)	38.1(37.7–39.0)	0.673
X-ray change	9(100.0)	16(100.0)	
Fever	8(88.9)	15(93.7)	0.6
Productive cough	8(88.9)	7(43.7)	0.062
Dry cough	1(11.1)	8(50.0)	0.008
Running nose	2(22.2)	5(31.25)	0.501
Pharyngalgia	2(22.2)	3(18.7)	0.609
Dyspnea	7(77.8)	8(50.0)	0.176
Malaise	3(33.3)	2(12.5)	0.23
Headache	1(11.1)	0	0.36
Chest pain	1(11.1)	1(6.2)	0.6
Muscle aches	1(11.1)	0	0.36
Abdominalgia	2(22.2)	0	0.12
Diarrhea	5(55.5)	2(12.5)	0.034

†BT(95%CI): Body Temperature (95% Confidence Interval). The statistic difference of age distribution between the two disease groups was tested by the Kruskal-Wallis chi-square test. Differences of gender and clinical symptoms were tested by the Fisher's exact chi-square test.

Characteristics and antibody level of P(H1N1) vaccinees and healthy control were demonstrated in Table 2. The two groups had similar age (p = 0.226) and gender (p = 0.572) distributions. The geomean of haemoglutinin inhibition (HI) antibody titer of P(H1N1) vaccinees was about 220.3 IU/ml, which was markedly higher than that of healthy controls (1.8 IU/ml, p<0.0001).

**Table 2:** Characteristics and antibody levels of P(H1N1) vaccinees and healthy subjects from year 2009 in Shanghai.

Characteristics	No. of healthy subjects (%)	No. of P(H1N1) vaccinees (%)	p Value
Study subjects	26	26	
Age (median; IQR)*	32(27–42)	39(30–46)	0.226
Male	11(42.3)	9(34.6)	0.572
Geomean of P(H1N1) HI Antibody titer (IU/ml)	1.8	220.3	0.000
IL-17 positive detection	3.8%(1/26)	Under detectable	
IL-23 positive detection	Under detectable	Under detectable	

*IQR: Inter Quantile Range. The statistic difference of age distribution between the two study groups was tested by the Kruskal-Wallis chi-square test; Difference of gender was tested by the Fisher's exact chi-square test. Data of antibody titer were log transformed, and difference of antibody titer from two groups was measured by T test.

### Sera cytokines of patients with mild symptoms

**Figure 1:**
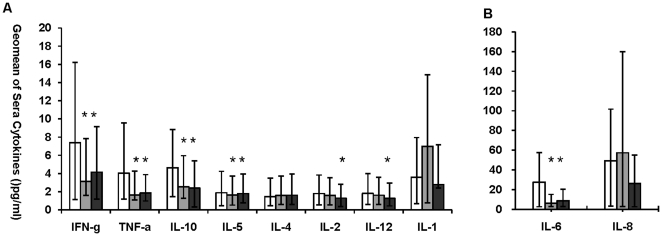
Sera cytokine levels of P(H1N1) patients, non-ILIs and healthy controls. Geomean production of IFN-γ, IL-1, IL-2, IL-4, IL-5, IL-10, IL-12, TNF-α, IL-6 and IL-8 expression in the sera samples from pandemic H1N1 influenza [P(H1N1)] (white bars, n = 77), non-flu influenza like illness (non-ILIs) (Light grey bars, n = 59) and healthy controls (Dark grey bars, n = 26) were graphed as figure 1A and 1B. Bars represent geomeans ±95% Confidence Interval (CI). * Indicates significant differences (p≤0.05) between P(H1N1) patients and non-ILIs patients, or between P(H1N1) patients and healthy controls.

Seventy seven P(H1N1) patients and 59 non-ILIs patients, both with mild symptoms, were included in this study, and 26 sera samples from health individuals were collected as healthy controls. The levels of sera IFN-γ, TNF-α, IL-1, IL-2, IL-4, IL-5, IL-6, IL-8, IL-10, IL-12, IL-17 and IL-23 were measured. Sera level of IL-8 in non-ILIs cases were significantly higher (p = 0.005) (Figure 1B) than that of healthy controls. Although the levels of sera IL-1, IL-12, and TNF-α, Th2 cytokine of IL-10 in non-ILIs patients were higher than those of healthy controls, no statistic differences were observed (Figure 1A). P(H1N1) patients secreted significantly higher level of sera Th1 cytokines of, IL-2 (p = 0.022), IL-12 (p = 0.049), IFN-γ (p = 0.013), IL-6 (p = 0.013) and TNF-α (p = 0.000), Th2 cytokine of IL-5 (p = 0.044) and IL-10 (p = 0.017) than healthy controls (Figure 1A and 1B). When compared with the data of the non-ILIs, P(H1N1) patients produced higher level of the sera Th1 cytokines of IFN-γ (p = 0.002), IL-6 (p = 0.000) and TNF-α (p = 0.003), Th2 cytokine of IL-5 (p = 0.005) and IL-10 (p = 0.000).

Since the sera levels of IL-17 and IL-23 were too low to be detected in most samples, we used the positive detection rate to present the data. The respective positive detection rate of IL-17 from P(H1N1) patients, non-ILIs cases and healthy controls were 19.5% (15/77), 16.9% (10/59) and 3.8% (1/26), respectively (Fisher exact chi-square test, p = 0.049). The respective positive detection rate of IL-23 of P(H1N1) patients, non-ILIs cases and healthy controls were 13.0% (10/77), 37.3% (22/59) and 0% (0/26) (MH chi-square test, p = 0.001), respectively. Among the positive samples, the IL-17 and IL-23 levels were higher in the P(H1 N1) patients than those of health controls.

These results suggested that P(H1N1) infection induced an early and comprehensive innate immune response with higher levels of Th1, Th2 and Th3 cytokines secretion.

### Sera cytokines of severe P(H1N1) patients and severe non-ILIs cases

**Figure 2:**
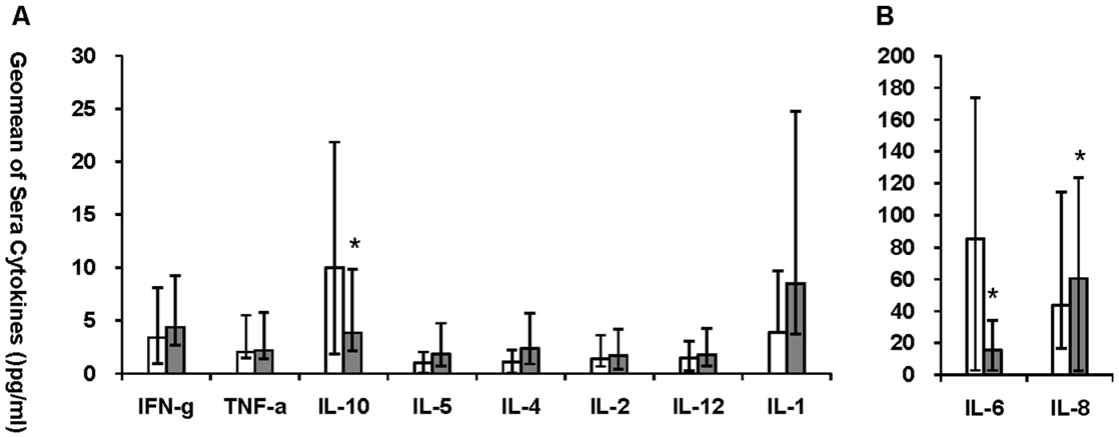
Sera cytokine levels of severe P(H1N1) and non-ILIs patients of 2011. Geomean production of IFN-γ, IL-1, IL-2, IL-4, IL-5, IL-10, IL-12, TNF-α, IL-6 and IL-8 expression in sera samples from severe P(H1N1) patients (White bars, n = 9) and severe non-ILIs (Grey bars, n = 16) were graphed as figure 2A and 2B. Bars represent geomeans ±95% CI. * Indicates significant differences (p≤0.05) between 2 study groups.

Nine severe P(H1N1) patients and 16 non-ILIs were from the hospitalized patients of year 2011. Compared with the severe non-ILIs patients, severe P(H1N1) patients displayed markedly increased sera production of IL10 (p = 0.032) and IL-6 (p = 0.048) respectively, and reduced production of IL-8 (p = 0.048). Statistic difference was not observed in the sera levels of IFN-γ, TNF-α, IL-1, IL-2, IL-4, IL-5 and IL-12 between the two groups (Figure 2A and 2B), as well as in IL-17 and IL-23.

These results indicated that the severe P(H1N1) patients secreted higher level of sera IL-6 and IL-10 than that of the severe non-ILIs patients,which is an indication of a unique pathogenicity of P(H1N1) infection.

### Sera cytokines of P(H1N1) vaccinees and healthy controls

**Figure 3:**
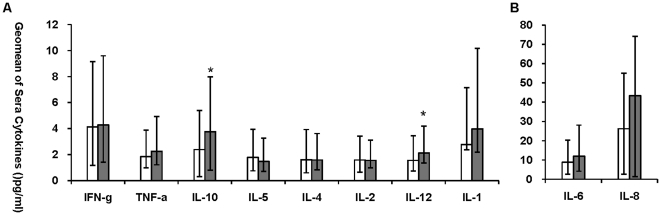
Sera cytokine levels of P(H1N1) vaccinees and healthy controls. Geomean production of IFN-γ, IL-1, IL-2, IL-4, IL-5, IL-10, IL-12, TNF-α, IL-6 and IL-8 levels in sera samples from P(H1N1) vaccinees (Grey bars, n = 26) and healthy controls (White bars, n = 26) were graphed as figure 4A and 4B. Bars represent geomeans ±95% CI.* Indicates significant differences (p≤0.05) between 2 study groups.

To identify the relationship between up-regulated cytokine levels and P(H1N1) infection, 26 sera samples with a hemagglutinin inhibition (HI) antibody titer for P(H1N1) higher than 40 IU/ml were collected 1 month after P(H1N1) vaccination, and 26 sera samples with a HI antibody titer for P(H1N1) lower than 40 IU/ml were randomly selected as healthy controls. Cytokines and chemokines levels in sera were measured in both groups. P(H1N1) vaccinees secreted markedly higher level of sera IL-12 (p = 0.050) and IL-10 (p = 0.018) than those of healthy controls (Figure 3A and 3B). There were no difference in sera Th1 cytokines (IFN-γ, IL-1, IL-2, IL-6 and TNF-α), Th2 cytokines (IL-4, IL-5) and chemokine IL-8 between P(H1N1) vaccinees and healthy controls. Except one case of low level positive detection of IL-17 among 26 healthy controls there were no positive detections for sera Il-17 and IL-23 in all other cases including the vaccinees and healthy controls.

These results indicated that sera IL-10 was elevated not only in those individuals infected with P(H1N1) virus with mild and severe symptoms, but also in those immunized with P(H1N1) vaccine, indicating the role of IL-10 in P(H1N1) infection.

### Disease severity and cytokine expression

**Figure 4:**
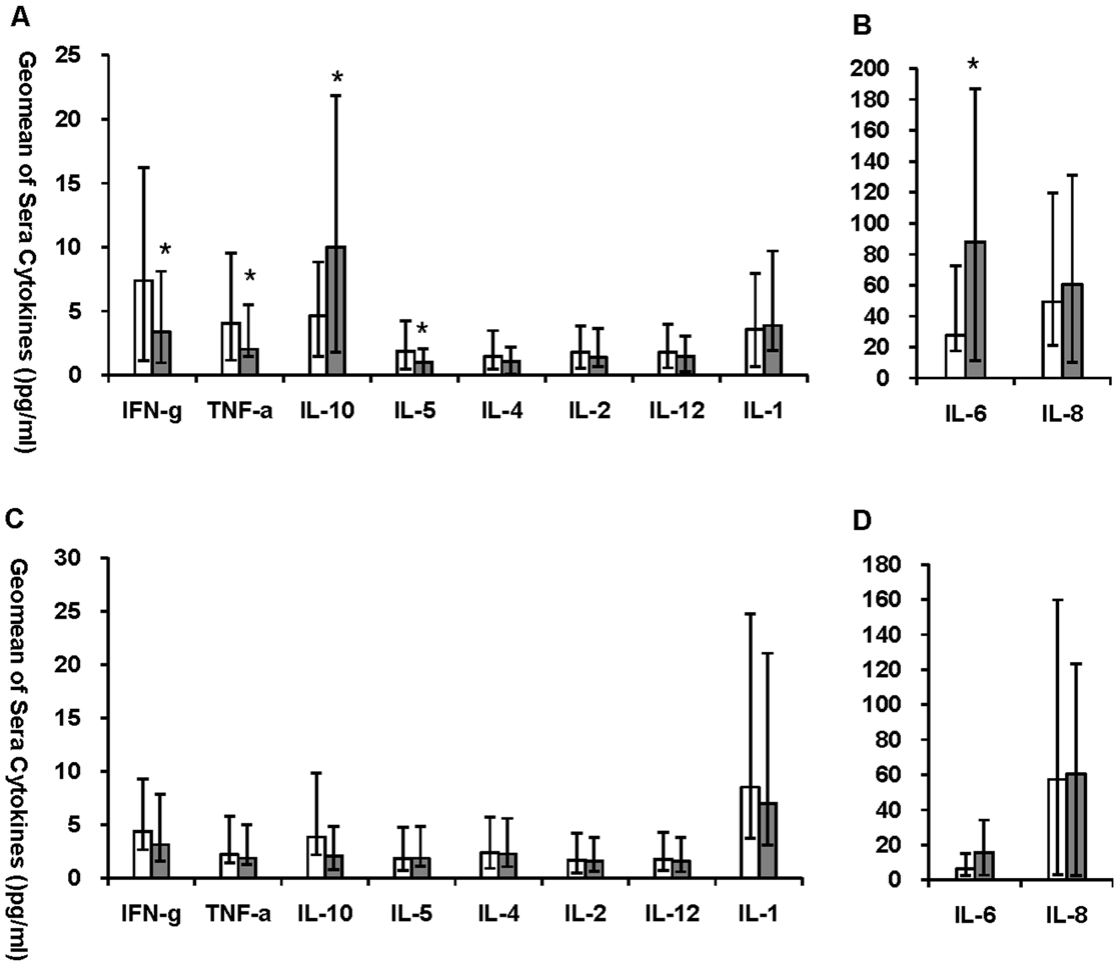
Sera cytokine levels between people got mild and severe infection. Geomean production of IFN-γ, IL-1, IL-2, IL-4, IL-5, IL-10, IL-12, TNF-α, IL-6 and IL-8 in sera samples from severe (Grey bars, n = 9) and mild P(H1N1) patients (White bars, n = 77) were graphed as figure 4A and 4B. Geomean production of IFN-γ, IL-1, IL-2, IL-4, IL-5, IL-10, IL-12, TNF-α, IL-6 and IL-8 in sera samples from severe (Grey bars, n = 16) and mild non-ILIs (White bars, n = 59) were graphed as figure 4C and 4D. Bars represent geomeans ±95% CI. * Indicates significant differences (p≤0.05) between 2 study groups.

To elucidate the association between cytokine production and disease severity, we analyzed the sera cytokine levels of patients with mild and severe symptoms in P(H1N1) and non-ILIs patients. Compared with the mild P(H1N1) patients, severe P(H1N1) patients produced markedly higher levels of IL-6 (p = 0.030) and IL-10 (p = 0.0006), but significantly lower levels of IFN-γ (p = 0.022), TNF-α (p = 0.033) and IL-5 (p = 0.001) (Figure 4A and 4B), respectively. No statistic difference was observed in sera levels of cytokines of IFN-γ, TNF-α, IL-1, IL-2, IL-4, IL-5, IL-6, IL-10, IL-12, IL-17 and IL-23 and chemokine IL-8 (Figure 4C and 4D) between non-ILIs patients with mild and severe symptoms. Thus, our results suggested that elevated sera levels of IL-6 and IL-10 may relate to the disease progression and severity in patients infected with P(H1N1).

### Sera levels of IL-6 and IL-10 and body temperature in P(H1N1) patients

**Figure 5:**
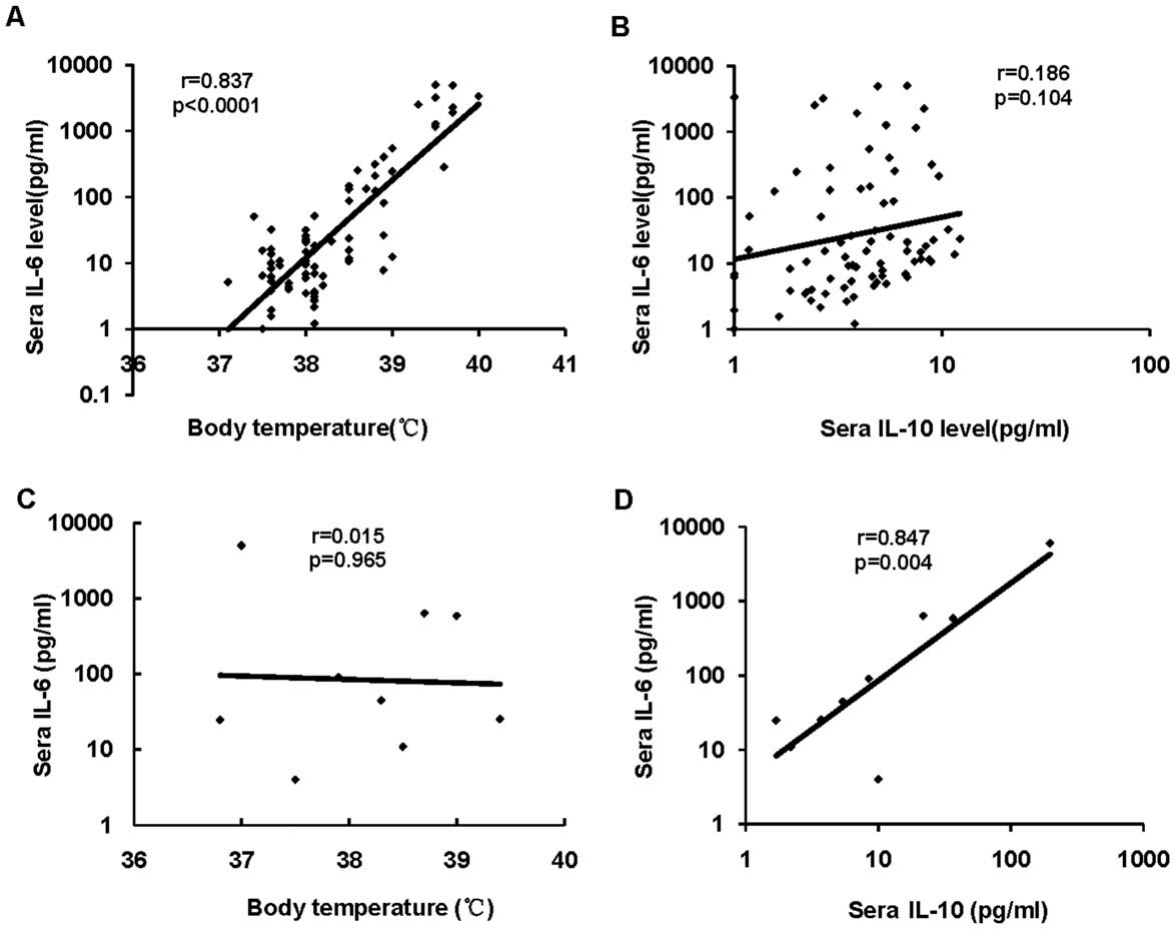
The associations between IL-6 level and body temperature, and between IL-6 and IL-10. Associations of the of body temperature (graph 6A) and production of IL-10 (graph 6B)) together with sera IL-6 levels in mild P(H1N1) patients from year 2009 were analyzed using Pearson correlation. Correlations of the of body temperature (graph 6C) and production of IL-10 (graph 6D)) together with sera IL-6 levels in severe P(H1N1) patients from year 2011 were analyzed using Pearson correlation. The “r” (correlation coefficient) and p values are indicated on respective graphs obtained from the correlation analysis.

Since IL-6 may have both pro- and anti-inflammatory effects on macrophages, and IL-10 is a potent inhibitor for macrophage function, we analyzed the association between IL-6 and IL-10 production in all P(H1N1) patients. In addition, the association between IL-6 level and body temperature was also analyzed. In the mild P(H1N1) patients from year 2009, we observed a positive correlation between patients' sera IL-6 level and body temperature with a correlation coefficient of 0.837, p<0.0001 (Figure 5A), but no correlation between patients' sera IL-6 and IL-10 with a correlation coefficient of 0.186, p = 0.104 (Figure 5B). In severe P(H1N1) patients, no correlation between sera IL-6 level and body temperature was observed with a correlation coefficient of 0.015, p = 0.965 (Figure 5C). However, a positive correlation between IL-6 and IL-10 levels was observed with a correlation coefficient of 0.847, p = 0.004 (Figure 5D) in these patients.

## Discussion

The innate immune system serves as the first line to fight against pathogens. Production of cytokines and chemokines is the first ramification of activation of the innate immune cells. The interleukins, together with IFN-γ, TNF-α and other chemokines, help regulate inflammation and the intensity of immune response, and play a role in activating the adaptive immune response [16].

Up-regulated levels of IL-1, IL-6, IL-12, and IL-23 were reported to be related to increased responses to infection, such as fever, phagocytic cell recruitment and blood vessel permeability. Increased cytokine production upon P(H1N1) infection was considered as an important player in the pathogenesis and disease development based on the following known observations. The invasion of P(H1N1) influenza virus into the lung tissues of mammals induces the production of pro-inflammatory cytokines and results in the development of pneumonia. In mice infected with P(H1N1) virus, the productions of IFN-γ, IL-4, IL-5, and IL-10 were increased. In macaques infected with P(H1N1), the levels of IL-6 and IL-18 were higher than those of non-infected ones [1]. In the homogenates of mouse lungs, the levels of the Th2 cytokines, notably IL-4, IL-5, and IL-10, were selectively induced after P(H1N1) infection [1]. In our current study, we collected sera from P(H1N1) patients to study the host innate immune response of the prodromal period to P(H1N1) infection. Comprehensive and strong cytokine induction in the early P(H1N1) infection with the higher level of sera IL-2, IL-12, IFN-γ, IL-6, TNF-α, IL-5, IL-10, IL-17 and IL-23 were observed.

Among the different cytokines analyzed in our study, the sera levels of IL-6 and IL-10 were considerably higher in the P(H1N1) patients than that of the non-ILIs infection patients and the healthy controls. We also observed that with the progression of clinical severity, sera levels of IL-6 and IL-10 in the study subjects were significantly up-regulated, with the lowest levels in the healthy controls, higher levels in the mild P(H1N1) patients and the highest levels in the severe P(H1N1) patients. In addition, the vaccinees who had HI antibody to P(H1N1) displayed markedly increased level of sera IL-10 compared to that of the healthy controls. Therefore, the roles of both IL-6 and IL-10 in P(H1N1) infection were indicated. As one of the important proinflammatory cytokines, IL-6 was elevated in patients with inflammatory diseases. IL-6 is induced by inflammatory stimuli, such as IL-1 and TNF-a [17]. IL-10 acts as a major immunomodulatory cytokine. It can inhibit antigen-presenting cells (APC) and macrophage function, suppress Th1 cytokine production and impair T-cell responses. IL-10 can also induce immune anerge [16] and differentiation of regulate T cell [18]. Previous study suggested that both IL-10 and IL-6 mainly act via the Janus kinases (Jaks) and STAT signal transduction pathway and may share some common biological activities [19]. IL-6 was identified as the inducer of IL-10 production to some extent [20]. They may function in concert to clear pathogens and regulate cellular immune responses, which are critical for the host to protect against lethal influenza, however, resulting in destructive tissue inflammation.

**Figure 6:**
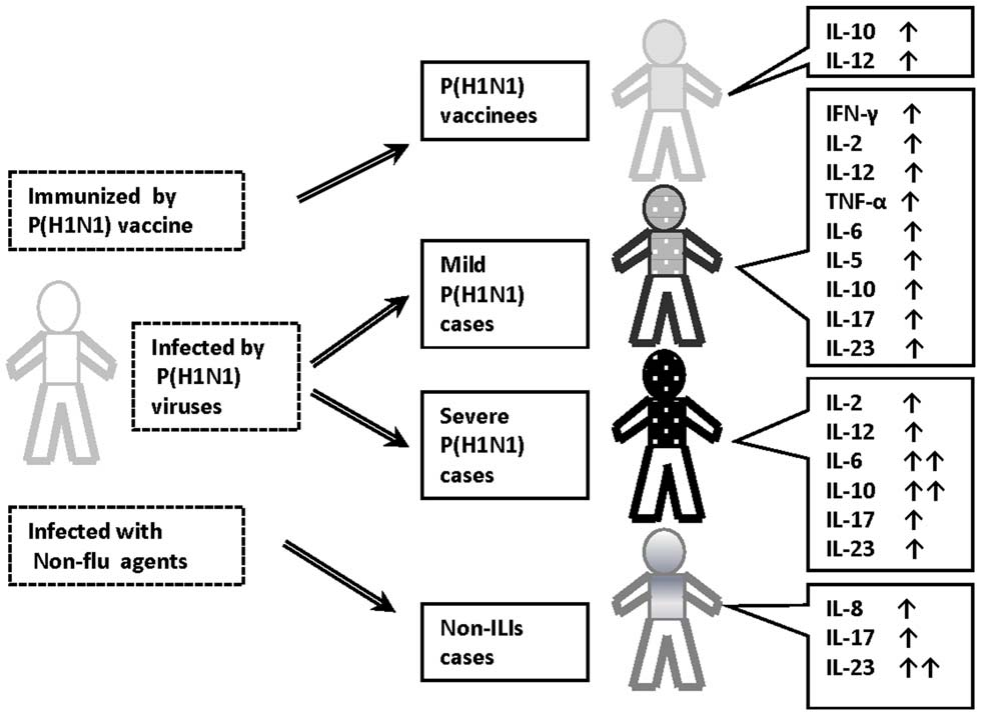
The schematic diagram summarizing the association observed in this study. The schematic diagram was designed to describe the innate immune response of individuals who have been immunized with the pandemic H1N1 influenza vaccine, infected with P(H1N1) virus, or infected with other non-influenza respiratory agents. The sera cytokines and chemokine secretion status serve as indicators of the innate immune response. ↑Indicates markedly up-regulated cytokine levels; ↑↑Indicates robust up-regulated cytokines levels.

The P(H1N1) patients from year 2009 were febrile travelers quarantined at the entry of Shanghai from outside of China. Most of them were still at the prodromal stage of the disease. During the initial response to P(H1N1) virus, the host macrophages may become activated and release pro-inflammatory cytokines to stimulate the immune response that leads to the symptoms of inflammation. This is evidenced by the fact that mild P(H1N1) patients suffered from higher fever (Figure 6A), elicited more potent production of pro-inflammatory cytokines such as IFN-γ, TNF-α and IL-6 (Figure 1A and 1B) than the non-ILIs cases. Therefore, the increased level of IL-6 in mild P(H1N1) cases (27.5 pg/ml ) compared to that of the healthy controls (8.8 pg/ml ) may relate to fever and the acute response since strong correlation between patients' sera IL-6 and body temperature was observed (Figure 6A). It is possible that with the progression of the disease, the anti-inflammatory cytokines were produced to limit the extent of the pro-inflammatory activities. This is supported by the observation that severe P(H1N1) patients did not experience higher fever than the severe non-ILIs. Up-regulated level of sera IL-6 in the severe P(H1N1) patients ( 10.0-fold, 88.0 pg/ml in severe cases in contrast to 8.8 pg/ml in the healthy ones) may mainly be caused by the activation of IL-10 production (4.2-fold, 10.01 pg/ml in severe cases in contrast to 2.39 pg/ml in healthy ones). This is evidenced by the strong correlation between sera levels of IL-6 and IL-10 (Figure 6D), together with the poor association between IL-6 production and body temperature (figure 6C) in severe P(H1N1) patients. IL-6 may also promote IL-10 function, as evidenced by the phenomenon that severe P(H1N1) cases produced significant lower level of sera pro-inflammatory cytokines, such as IFN-γ (p = 0.022), TNF-α (p = 0.033) and IL-5 (p = 0.001), as compared to the mild patients. Furthermore, we found that the severe P(H1N1) patients more frequently experienced productive cough and diarrhea than the severe non-ILIs. However, more severe non-ILIs suffered from dry cough than the severe P(H1N1) ones (Table 1). This raised the possibility of viral persistence or secondary infection resulted from immunosuppression mediated synergistically by IL-6 and IL-10. Our results are consistent with the results from a study using an animal model infected with seasonal influenza A virus [21]. They found that the production of IL-10 did not impact virus clearance and disease recovery at a low virus dose. Instead, production of IL-10 was detrimental and dramatically reduced mice survival during lethal-dose virus challenge [21]. The symptoms and fever correlated with the release of IL-6 both in experimental models and patients with naturally acquired acute seasonal influenza A were observed in other studies. The up-regulation of the sera IL-10 level suggested a higher chance of disease persistence or secondary opportunistic infection in P(H1N1) infection. Thus the immunosuppressive environment created by IL-10 in synergism with IL-6 may lead to the virus persistence or secondary infection, which may result in the more severe symptoms in the severe P(H1N1) patients. In our present study, the 9 severe P(H1N1) patients are all children. It is known that the innate immune system functions differently in children as compared to adults. Therefore, more studies are needed for better insights. Despite of this our results still provide new understanding of the P(H1N1) pathogenesis, and warrant further investigations in adult severe P(H1N1) patients.

In a study of cytokine profile of P(H1N1) infection, Österlund et al [8] and his colleagues used a typical North American/European lineage of P(H1N1) virus to infect human monocyte-derived DCs and macrophages *in vitro*. Their results showed that P(H1N1) induced a relatively weak innate immune response in these cells. Moreover, Mukherjee et al [9] studied the host gene expression response to an Indian isolate of P(H1N1) infection in A549 cells using a microarray platform. They found that P(H1N1) infection resulted in considerable downregulation of the expression of cytokines, namely IL-8, and IL-4, compared to seasonal H1N1. A resent research on cytokine levels in P(H1N1) infected humans was performed in Romania [22]. They detected IL-6, IL-8, IL-12, IL-17, TNF-a and IFN-γ in both mild and severe P(H1N1) patients hospitalized with healthy control individuals. They reported significantly higher levels of sera IL-6, IL-12, IP-10 and TNF-a in the mild P(H1N1) patients than those of healthy controls,. different from our observation of a comprehensive innate immune response in mild P(H1N1) patients with up-regulated levels of sera IL-2, IL-12, IFN-γ, IL-6, TNF-α, IL-5, IL-10, IL-17 and IL-23. The difference might be due to the sample types between Romania study and ours. First the age median of the Romania mild P(H1N1) patients was 33 years old (IQR,18 to 35), and 64% (7/11) of them had other diseases, such as cardiovascular disease, asthma/COPD, obesity and cancer. While the age median in our study was 16 (IQR, 21–27), and, 6% (5/77) mild P(H1N1) patients had underlying diseases. The other diseases in Romania mild P(H1N1) patients may impact their innate immune response to some extent and affected their cytokine profiles. Second, the sera collection time after P(H1N1) infection might also contribute the difference. We collected the patient sera sample at the date of fever onset (during the prodromal period of disease), while the sera samples in Romnia were collected 2 days (IQR 2 to 3) after the onset of illness, which is the host innate immune response in a later stage of the disease. Previous *in vitro* study observed that T-cell activation and proliferation genes expression increased at the early stages, but no changes at the later stages in P(H1N1) infected human DC [9]. Third, in the Romania study, there were only 11 mild and hospitalized (more serious clinical symptoms) patients. In our present study, a total of 77 mild P(H1N1) patients were investigated. Taken together, all the above factors may have affected the different cytokine profiles between the Romania patients and those in our study.

### Concluding remarks

Our observations provide new evidence that a comprehensive innate immune response is activated at the early stage of pandemic H1N1 influenza virus infection with up-regulated production of sera IL-2, IL-12, IFN-γ, IL-6, TNF-α, IL-5, IL-10, IL-17 and IL-23. With disease progression, higher levels of sera IL-6 and IL-10 were found in severe pandemic H1N1 influenza patients. We further identified a strong correlation between IL-6 and IL-10 production in the severe P(H1N1) patients. Significantly elevated IL-10 secretion in the P(H1N1) vaccinees further confirmed our conclusion that highly elevated levels of sera IL-6 and IL-10 in P(H1N1) patients may lead to disease progression and severity. And the increased IL-6 and IL-10 levels may together be the underlying cause of the observed clinical severity in the severe P(H1N1) patients. In-depth underlying mechanisms await further detailed investigations in the future. The findings of this study are concluded into Figure 6.

## Materials and Methods

### Study Subjects

#### 1) Patient with mild symptoms

In Shanghai, the instituted health monitoring by temperature screening was implemented in 2009 at all ports of entry for travelers arriving from areas or countries heavily affected by pandemic H1N1 virus. According to the requirement of “*Interim Guidance on Infection Control Measures for 2009 H1N1 Influenza*” issued by the Chinese Center for Disease Control and Prevention (CCDC) [23], any travelers who were experiencing acute respiratory illness and fever (body temperature≥37.5 degrees Celsius) were isolated from the healthy population. The throat/nose swab and blood samples of the illed travelers were taken based on informed consent. All samples were sent to the microbiology laboratory of Shanghai Municipal Center for Disease Control and Prevention (SCDC) for influenza virus detection. Subsequently, experienced public health staff interviewed case patients by a standardized questionnaire to determine symptoms at the time of sample collection. This information was entered into SCDC database. All samples were analyzed for influenza A, B and subtypes of A by influenza real-time RT-PCR test. A total of 77 serum samples from patients with confirmed P(H1N1) infection and 59 serum from non-ILIs patients were taken. Both groups were of mild symptoms.

#### 2) Patients with severe symptoms

In 2011, a total number of 25 throat swabs and blood samples from hospitalized pneumonia patients were sent to our laboratory by their parents for influenza analysis. Nine of them were positive for P(H1N1) detection, and the rest 16 negative for human influenza A and B virus detection by influenza real-time RT-PCR test.

#### 3) P(H1N1) vaccinees and healthy study subjects

According to the Immunization Strategy [24] issued by CCDC, from September 15, 2009, public servants at key positions (e.g., health staff), being the high priority populations, were administered with the P(H1N1) vaccine under the principles of inform consent, voluntary and free of charge. Before vaccination and after vaccination (1, 6 and 12 months post), blood samples were collected from the randomly selected subjects who accepted immunization from their county CDCs [25]. Case demographic and epidemiological information was investigated at the same time of sample collection. All blood samples from Shanghai were transported to and stored in the *SCDC Mass Vaccination Surveillance Sample Bank* (SCDC MVSSB) for analysis. We randomly selected 26 sera samples before vaccination with a Hemagglutinin Inhibition (HI) antibody titer for pandemic H1N1 virus lower than 40 IU/ml as healthy controls, and 26 sera samples 1 month after vaccination with a HI antibody titer for pandemic H1N1 virus higher than 40 IU/ml for the P(H1N1) vaccinees.

### Influenza Real-time RT-PCR

The total nucleic acid of each swab (200 µl) was isolated using the MagNA Pure LC 2.0 Instrument (Roche Diagnostics Ltd, Switzerland) and a MagNA Pure LC DNA Isolation Kit (Roche Diagnostics GmH, Germany) following manufacturer's recommendations. Sixty micro liter of eluted and purified DNA was stored at 4°C and used as templates for PCR within 4 hours. Influenza Real-time RT-PCR was performed in all swabs using primers and probes described in the CCDC A(H1N1) influenza guidance.

### CBA Assays

Sera IL-2, IL-4, IL-5, IL-10, TNF-α and IFN-g levels were evaluated using the cytokine bead array (CBA) and the Human Th1/Th2 Cytokine CBA kit(BD Biosciences, San Jose, CA, USA). IL-1, IL-6, IL-8, IL-12 levels were assayed using a Human Inflammatory Cytokine CBA kit (BD Biosciences, San Jose, CA, USA). Bead populations with distinct intensities coated with capture antibodies specific for each cytokine and chemokine were mixed and added to each assay tube, followed by Phycoerythrin (PE) detection reagent and plasma. Mixtures were incubated at room temperature for 3 hrs in darkness, washed, and then analyzed using a flow cytometry (Falcon; BD Biosciences). Data were analyzed using the BD CBA analysis software. The individual cytokine concentration of each test sample was calculated against the reference to a standard curve. Data were log transformed, and geomeans ±95%CI are shown in the figures.

### Cytokines ELISA

Sera IL-17 and IL-23 levels were analyzed using a commercial Human Il-17 ELISA Kit and the Human IL-23 ELISA Kit (ABCAM, US). Experiments were performed according to the manufacturer's recommendations. IL-17 and IL-23 levels were determined by comparison with a standard curve. Cytokine levels were measured as duplicates and averaged.

### Statistical Analyses

All data were processed using SPSS for Windows version 13.0. Differences in age ratio among different disease groups and body temperature were analyzed using the nonparametric Kruskal-Wallis chi-square test. Gender distribution was analyzed using the Pearson chi-square test. Since the data of cytokine levels were highly variable, the values were log transformed. The geomean and its 95% CI were then calculated and independent t-test was performed to detect the differences based on the log transformed data. Statistic significance was considered when p value is equal or less than 0.05.
